# Effects of Rosmarinus officinalis and Platanus orientalis extracts on asthmatic subjects resistant to routine treatments

**Published:** 2018

**Authors:** Majid Mirsadraee, Afsaneh Tavakoli, Vahideh Ghorani, Shadi Ghaffari

**Affiliations:** 1 *Department of Internal medicine, Faculty of Medicine, Islamic Azad University-Mashhad Branch, Mashhad, Iran*; 2 *Innovative Medical Research Center, Department of pharmacology,* *Mashhad Branch, Islamic Azad University, Mashhad, Iran.*; 3 *Neurogenic Inflammation Research Centre and Department of Physiology, Faculty of Medicine, Mashhad University of Medical Sciences, Mashhad, Iran*; 4 *Biologist, Shahid Hasehemi Nezhad Research center, Kavosh high school, Ministry of Education, Mashhad, Iran*

**Keywords:** Asthma, R. officinalis, P. orientalis, Sycamore, Rosmarinic acid

## Abstract

**Objective:**

The present study aimed to determine the effects of *Rosmarinus officinalis *(*R. officinalis)* and *Platanus orientalis* (*P. orientalis*) extracts on asthma.

**Materials and Methods:**

We conducted a randomized, double-blind, active-comparator study to evaluate the effect of *P. orientalis* and *R. officinalis* extracts on asthmatic patients resistant to routine treatment. The subjects were randomly divided into three groups receiving *P. orientalis* and *R. officinalis* extracts alone or in combination. The primary endpoints were clinical findings, spirometry, exhaled nitric oxide (FENO) and Asthma Control Test (ACT) assessed over the one-month treatment period.

**Results:**

ACT score showed significant improvement after treatment with *R. officinalis* (p<0.05) but not with *P. orientalis*. Clinical evaluations showed that cough, sputum production and wheezing were significantly improved in *R. officinalis* group (p<0.05 to p<0.001) while in *P. orientalis* group only improvement of cough and chest tightness were shown. Spirometry results showed significant improving in FEV1/VC values for subjects treated with *P. orientalis *and those who received the combination of extracts as well as significant decrease in FEF25-75 value only for *P. orientalis* group (p<0.05 for all cases). FENO was decreased in both groups but the results were statistically significant only for *R. officinalis* group (p<0.05). Abdominal pain and skin rash were the most frequent side effects of the treatments which led to discontinuation of the intervention.

**Conclusion:**

*R. officinalis* extract showed promising results in treatment of resistant asthma. Further studies to find the most effective components of these herbal medicines are recommended.

## Introduction

Nowadays, asthma is being uniformly managed by the GINA guideline but still some subjects may show resistance to step four of GINA treatment guideline. In these subjects new not-established treatment may be used which could be driven from the traditional medicine. Platanus (*Platanus orientalis*) and Rosemary (*Rosmarinus officinalis*) extracts alone or in combination, were used in traditional Iranian medical sciences for a long time. 

*Platanus orientalis *(*P. orientalis*) (Plane tree, ČENĀR (Encyclopedia Iranica, 2014 ▶), Sycamore) is a medicinal tree from Platanaceae family (Hajhashemi et al., 2011 ▶) which grows from south-eastern Europe to northern Persia and has known to induce allergic reactions such as asthma in our region (Pazouki et al., 2008 ▶) by a cyclophilin family molecule (Pazouki et al., 2009 ▶). Surprisingly, its extract was traditionally used for treatment of asthma and is widely available in herbal medicine stores in Iran, but no scientific evidence about this effect is available in modern medicine. This plant contains flavonoids such as quercetin, kaempferol and their glycosides that have anti-inflammatory effects (Mitrokotsa et al., 1993 ▶; Chirumbolo, 2010 ▶).

*Rosmarinus officinalis* (*R.* *officinalis*) extract is another drug which is used by Iranian herbalist as an admixture with *P. orientalis* extract. *R.* *officinalis* grows in many parts of the world and has several pharmacological effects (Mehni et al., 2012 ▶; Swarup et al., 2007 ▶). It is used as a relieving agent in respiratory disorders and has relaxant effects on tracheal smooth muscles (Al-Sereiti et al., 1999 ▶). Anti-inflammatory effect of *R. officinalis* extract was also reported (Emami et al., 2013 ▶; Bai et al., 2010 ▶). The effect of *R. officinalis* extract on asthma and its mechanisms was more investigated compared to *P. orientalis*. The most important component of *P. orientalis* is caffeic acid and its derivatives, rosmarinic acid which is able to inhibit the production of leukotriene B4 in human polymorphonuclear leucocytes (Al-Sereiti et al., 1999 ▶) and ursolic acid which inhibits the nuclear transcription factor kappaB (nfK-B) (Aggarwal and Shishodia, 2004 ▶). Oxidative molecules over-production and lesser antioxidative activity are also another problem which may arise in asthma (Sahiner et al., 2011 ▶). Eftekhar et al. (2017) ▶ showed beneficial effect of high titer of rosemarinic acid against oxidative stress in an experimental model of asthma. According to beneficial effects of *R. officinalis* on two most important mediators of asthma, it should be addressed that if *P. orientalis* and *R. officinalis* could be used for treatment of asthma, at least in addition to standard treatments. The objective of this study was to determine the effect of *P. orientalis* and *R. officinalis* extracts alone or in combination, for treatment of resistant asthma.

## Materials and Methods

**Subjects**

Forty four subjects (17 male and 27 female) were enrolled in this study. Eligible subjects were previously experienced a long-term standard treatment of asthma including inhaled corticosteroid, long-acting beta-2 agonist, antileukotriene and theophylline according to GINA guidelines (Obyerns, 2006), but still clinically and physiologically resist to these treatments and had experienced episodes of exacerbation. Exclusion criteria included: 1- Respiratory infection 2- Fair treatment with controller drugs 3- Not cooperative with project 4- Congestive heart failure 5- Gastroesophageal reflux 6- Rhinosinusitis, 7- Pregnancy and 8- Smoking.

**Study configuration and methods**

This study was an active-comparator clinical trial in which previous asthma therapy was continued. The design of study was a cross over, randomized, double-blind controlled clinical trial performed at subspecialty clinic of lung disease during 2012-2013. The study was approved by Ethics Committee of Islamic Azad University-Mashhad branch and followed by COPD research center (Ethics approval code: A34-18) and was also registered in Iranian Registry of Clinical Trials (IRCT Code: IRCT201107312695N2). Before beginning the study, benefits of these herbal drugs were explained to subjects and all patients provided written informed consent. Patients were permitted to continue their previous treatment (ICS and LABA combination, Montelukast and Theophylline). 

In this three-phase trial, all subjects were randomly divided in two groups. In the first phase, group 1 received *P. orientalis* leaves extract in addition to previously prescribed drugs and group 2 received *R.* *officinalis* leaves extract for one month. Variables that were evaluated before administration of the herbal drugs, consisted of clinical parameters including symptoms (cough and dyspnea) and sign (wheeze) and clinical improvement of asthma validated by Asthma Control Test (ACT) questionnaire (ACT which is a valid questionnaire for evaluating the activity of asthma), and paraclinical tests including spirometry (Superspiro, Micromedical instruments, England) and Exhaled Nitric Oxide (FENO) (No Breath, Bedfont Medical instruments, England). Spirometry was done according to American Thoracic Society/European Respiratory society guidelines (Miller, 2005 ▶).

Primary endpoints were improvement of cough, dyspnea and the change from baseline in the total score of the ACT questionnaire that were all assessed over the one-month treatment period of each steps. 

Secondary endpoints were FEV1, FEV1/FVC and FENO. *R.* *officinalis* and *P. orientalis* leaves extract and placebos were produced by Birjand herbal company, Birjand, Iran. The patients daily received 50 ml herbal drugs as liquid (each one ml contained 200 mg herbal extract). Patients in each treatment group consumed the prepared extracts, 3 times a day before meal for one month. Doses of herbal drugs were selected based on previous studies (Pengelly et al., 2012 ▶; Rasoolijazi et al., 2015 ▶; Ulbricht et al., 2010 ▶; Hajhashemi et al., 2011 ▶; Irtiza et al., 2016 ▶). The herbal drugs were labaled by an independent physician and prescribed by another pharmacist who was blinded to lables. Patients and physicians were blind to the study group assignment throughout the study. After one month, the outcomes were evaluated again and subjects who showed complete improvement or side effects of the extract, were excluded from study. In order to prepare the subjects for another herbal drug, all subjects underwent a wash-out period and received placebo, similar to the previous drugs, for one month. 

In the second phase, the subjects in group 1 received *R.* *officinalis* leaves extract and group 2 received *P. orientalis* leaves extract for one month. Variables were measured before and after this step and then, all subjects experienced another wash-out period. 

**Figure 1:**
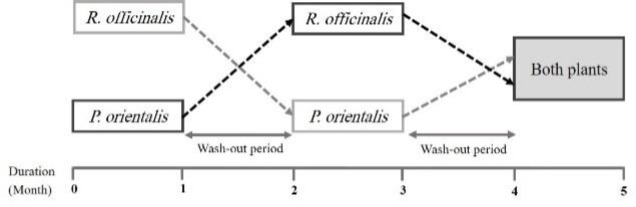
Diagram of the study design.

In the third phase (i.e. the fifth month), all subjects received both herbal drugs and were evaluated (Figure 1). Phone call follow-up was performed every 2 weeks and in case of acute attack, increasing the dose of inhaled corticosteroids (ICS) or one-week oral prednisolone was permitted.

**Statistical analysis**

In this study, data was represented as mean±SD. Sample size was calculated according to the prevalence of resistant asthma in our clinic, with 0.05 alpha errors and 80% potency. Comparison between the results of *P. orientalis* and *R. officinalis* groups were made by chi square and student t tests. The results of treatment during each phase, were analyzed by McNemar’s and paired t tests. SPSS 19 software was used for statistical analysis. Significance was accepted at p<0.05.

## Results

Twenty-three subjects of *R. officinalis* group experienced two phases and 21 subjects were present in *P. orientalis* group (Table 1). Fourteen subjects received both herbal drugs. Mean duration of asthma was 114±88 months which was not significantly different between two groups (t=1.13, p=0.2). From these subjects, 17 subjects completed the courses of *R. officinalis* and *P. orientalis* treatment and 12 subjects completed the course of mix treatment. Clinical findings, spirometry parameters, ACT score and FENO before trial were compared between the groups and no significant differences were observed. Six subjects (three subjects from each group) experienced side effects (Skin rash, and gastrointestinal intolerance) and quit the study. Overall, subjective improvement and patient satisfaction were seen in 14 subjects of *R. officinalis* group, 7 subjects of *P. orientalis* group and one subject of mixed-treatment group. Although ameliorative effects of *R. officinalis* were 2 times higher than that of the other treatment, but the statistical difference was not significant (X^2^=2.7, p=0.2). Side effects were not significantly different between groups and overall satisfaction was not significantly different between male and female patients.

**Table 1:** Demographic and basic data of resistant asthmatic subjects recruited for this clinical trial.

	**Total**	***R. officinalis***	***P. orientalis***
**Entered the study**	44	23	21
**Leaved the study**	10	6	4
**Completed the study**	34	17	17
**Age (Years)**	52±15 (18-87)	49±15	56±13
**Sex (Female/Male)**	27/17	13/10	11/10
**Duration of asthma (months)**	114±88	97±76	118±88
**Side effect**	6	3	3
**Overall improvement**	21	14	7

Data were presented as mean±SD.

**Comparison of clinical findings and spirometry parameters before and after treatment with** ***R.*** ***officinalis *and* P. orientalis* extracts**

**Figure 2:**
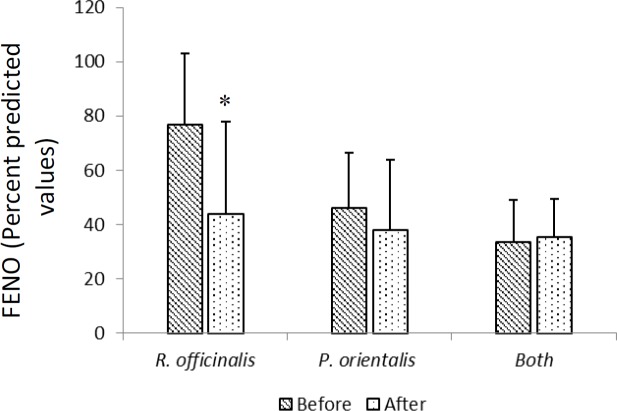
FENO value before and after-treatment in groups treated with *R. officinalis* and *P. orientalis* (n=17 in each group) and both extracts, (n=12) for one month. Values are expressed as mean±SD.*p<0.05 as compared to before treatment value within each group. Statistical analyses were performed using paired sample t-test. FENO: fractional exhaled nitric oxide

**Figure 3:**
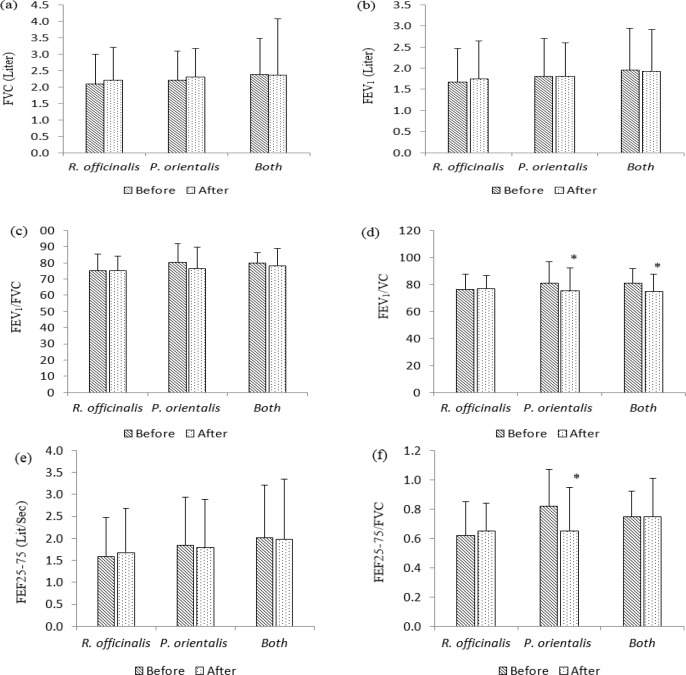
PFT values including FVC (a), FEV_1_ (b), FEV_1_/FVC (c), FEV_1_/VC (d), FEF25-75 (e) and FEF25-75/FVC (f) before and after-treatment in groups treated with *R. officinalis* and *P. orientalis* (n=17 in each group) and both extracts (n=12), for one month. Values are presented as mean±SD. *p<0.05 as compared to before treatment value within each group. Statistical analyses were performed using paired sample t-test.

FVC: forced vital capacity; FEV_1_: forced expiratory volume in first second; VC: vital capacity; and FEF25-75: forced expiratory flow at 25-75% of vital capacity.

**Figure 4:**
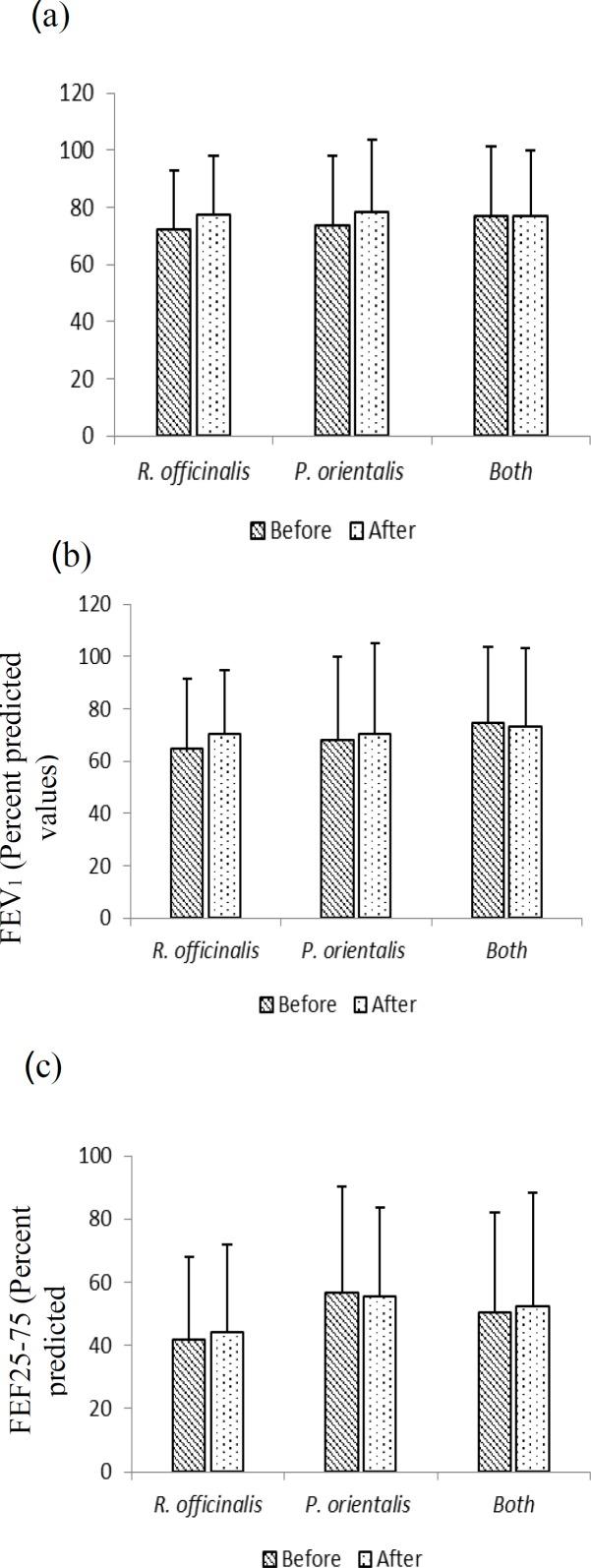
PFT values including FVC% (a), FEV_1_ %(b) and FEF25-75% (c) before and after-treatment in groups treated with *R. officinalis* and *P. orientalis* (n=17 in each group) and both extracts (n=12), for one month. Values are expressed as mean±SD. Statistical analyses were performed using paired sample t-test.

*R.* *officinalis* treated group showed significant improvement in clinical symptoms including cough (decreased by 37%), sputum (decreased by 48%) and wheezy chest reported by patients (decreased by 31%) compared to baseline values (p<0.05 to p<0.001). Dyspnea and wheezing on physical examination were decreased after treatment with *R.* *officinalis* but the difference was not significant (Table 2). Asthma was improved in terms of ACT score which increased significantly (p<0.05, Table 2) and asthma attack which non-significantly decreased (decreased by 16%). Spirometry parameters showed no significant changes but FENO was significantly decreased after treatment with *R.* *officinalis* (p<0.05, Figures 2-4). In group treated with *P. orientalis*, no significant changes were observed in clinical (Table 2) and inflammatory (FENO) parameters (Figure 2), while spirometry parameters such as FEV1/VC and FEF25-75/FVC were significantly decreased after treatment with *P. orientalis *extract compared to baseline values (p<0.05, Figure 3).

**Table 2:** Comparison of clinical findings of asthmatic subjects before and after treatment with *R. officinalis* and *P. orientalis* and both extracts.

**Clinical findings**	***R. officinalis*** **Before**	***R. officinalis*** ** After**	***P. orientalis *** **Before**	***P. orientalis *** **After**	**Both extracts** **Before**	**Both extracts** **After**
**Cough**	24 (70%)	11 (33%)***	21 (63%)	18 (53%)	9 (75%)	5 (45%)
**Sputum**	24 (70%)	4 (22%)***^+++^	16 (47%)	19(56%) ###	7 (58%)	3 (27%)**
**Dyspnea**	26 (76%)	20 (60%)	23 (68%)	21 (62%)	10(83%)	8 (64%)
**Chest tightness**	6 (19%)	10 (28%) ###	9 (25%)	4 (13%)	3 (22%)	1 (9%)
**Wheezy chest**	24 (71%)	14 (40%)*^+^	25 (73%)	26 (75%) ###	6 (50%)	3 (27%)
**Wheezing on physical exam**	29 (84%)	25 (73%)	30 (89%)	26 (75%)	8 (70%)	9 (72%)
**Asthma attack**	7 (20%)	2 (6%)	7 (21%)	7 (20%) #	3 (27%)	1 (9%)
**ACT score**	14.8±7.4	18.2±5.5*^+^	16.9±5.9	16.3±5.7	15.8±7.2	16.8±7.02

Statistical analyses were done by McNemar’s test and paired t test. Data were presented as mean±SD. Comparison between before and after treatment in each group:

Comparison of *R. officinalis* and *P. orientalis* groups vs both extracts group:

* p<0.05,

** p<0.01 and

*** p<0.001. Comparison between *R. officinalis* and *P. orientalis* groups:

+ p<0.05 and

+++ p<0.001

# p<0.05 and

### p<0.001

**Comparison of clinical findings and spirometry parameters before and after treatment with** ***R. officinalis*** **and** ***P. orientalis*** **combination**

One month treatment with *R. officinalis* and *P. orientalis* combination in the last phase of treatment showed significant improvement in sputum (p<0.01, Table 2) and no statistically significant decrease in cough and dyspnea. Additionally, there was no significant difference in ACT score, FENO and spirometry parameters except for FEV1/VC (p<0.05) after treatment with *R. officinalis* and *P. orientalis* combination compared to baseline values (Figures 2-4 and Table 2).

**Comparison between** ***R. officinalis*** **and** ***P. orientalis*** **groups**

After the course of treatment, significant improvements in terms of sputum production and wheezy chest were reported by patients in *R. officinalis* group compared to *P. orientalis* group (p<0.05 to p<0.001, Table 2). In addition, the ACT score in *R. officinalis* group was significantly higher than that of *P. orientalis* group (p<0.05, Table 2). The differences in other clinical findings, spirometry parameters and FENO were not significantly different among groups (Figures 2-4). 

FVC: forced vital capacity; FEV_1_: forced expiratory volume in first second; and FEF25-75: forced expiratory flow 25-75.

## Discussion

In this randomized controlled clinical trial, *R.* *officinalis* and *P. orientalis* leave extracts were used to treat asthmatic patients resistant to routine treatment based on the approached suggested by Iranian traditional medicine. Active comparator study was chosen to continue the previous therapy; in this cross-over study, all subjects were treated with each herbal medicine alone and finally received a combination of both. Most of the subjects were in their 50s and 60s and the average duration of asthma was ten years. According to GINA guidelines, they were in step five which means incomplete response to previous asthma therapy including four major anti-asthma drugs. Three subjects (15%) in each group showed side effects such as abdominal pain and skin rash to herbal drugs and discontinued the treatment. Some of the side effects of *R. officinalis* especially allergic contact dermatitis have been reported in previous studies (Ulbricht et al., 2010 ▶). 

The results of the present study indicated that cough, sputum, wheezy chest and ACT score were significantly improved in subjects treated with *R. officinalis* while improvements of cough and chest tightness were not statistically significant in *P. orientalis* treated group. The combination of the herbs only significantly decreased sputum. These findings suggest *R. officinalis* alone is more effective than *P. orientalis* (14 subjects showed signs of improvements in *R. officinalis* group compared to 7 subjects in *P. orientalis* group).

Experimental studies about *P. orientalis* and *R.* *officinalis* are scares. In a study, the relaxant effect of *R.* *officinalis* oil on tracheal smooth muscle was examined. The results indicated that inhalation of *R.* *officinalis* oil led to inhibitory effect on the contractions of rabbit and guinea pig tracheal smooth muscle induced by acetylcholine and histamine (Aqel, 1991 ▶). Some studies also reported anti-inflammatory effects of *R.* *officinalis *and *P. orientalis *(Emami et al., 2013 ▶; Chirumbolo, 2010 ▶). In the present study, decreased FENO (one inflammatory parameter) after treatment with *R.* *officinalis* and *P. orientalis *also demonstrated the probable anti-inflammatory effect of this plant.

Two mechanisms were explained for anti-inflammatory effect of *R.* *officinalis* (Al-Sereiti et al., 1999 ▶; Aggarwal and Shishodia, 2004 ▶). One attributes this effect to the presence of rosmarinic acid (one of the components of* R.* *officinalis*) which has antioxidant and anti-inflammatory properties and is able to inhibit the production of leukotrienes (Aggarwal and Shishodia, 2004 ▶; Stansbury, 2014 ▶). In a model of allergic asthma, rosmarinic acid was able to decrease the accumulation of inflammatory cells around vessels and bronchoalveolar lavage in murine sensitized to house dust mite (Inoue et al., 2005). Ursolic acid as another component of *R.* *officinalis* suppresses nuclear transcription factor kappaB (Aggarwal and Shishodia, 2004 ▶). This mechanism is able to suppress inflammatory reactions by inhibition of nuclear response of asthma. 

 In addition, inhibitory effect of rosmarinic acid on oxidative stress has been shown in experimental models which could also play a crucial role in the treatment inflammatory diseases such as asthma (Eftekhar et al., 2017 ▶). 

In conclusion, *R.* *officinalis* and rosmarinic acid have the potential to treat asthma but *P. orientalis* did not show any beneficial effect against asthma and probably its use for treatment of asthma should not be considered. 
